# p16Ink4a Prevents the Activation of Aged Quiescent Dentate Gyrus Stem Cells by Physical Exercise

**DOI:** 10.3389/fncel.2019.00010

**Published:** 2019-02-07

**Authors:** Laura Micheli, Giorgio D’Andrea, Manuela Ceccarelli, Alessandra Ferri, Raffaella Scardigli, Felice Tirone

**Affiliations:** ^1^Institute of Cell Biology and Neurobiology, National Research Council, Foundation Santa Lucia, Rome, Italy; ^2^Department of Ecological and Biological Sciences, University of Tuscia, Viterbo, Italy; ^3^Institute of Translational Pharmacology (IFT), National Research Council, Rome, Italy

**Keywords:** adult neurogenesis, dentate gyrus, neural stem cells, self-renewal, aging, physical exercise, p16Ink4a

## Abstract

In the neurogenic niches—the dentate gyrus of the hippocampus and the subventricular zone (SVZ) adjacent to lateral ventricles—stem cells continue to divide during adulthood, generating progenitor cells and new neurons, and to self-renew, thus maintaining the stem cell pool. During aging, the numbers of stem/progenitor cells in the neurogenic niches are reduced. The preservation of the neurogenic pool is committed to a number of antiproliferative genes, with the role of maintaining the quiescence of neural cells. The cyclin-dependent kinase inhibitor p16Ink4a, whose expression increases with age, controls the expansion of SVZ aging stem cells, since in mice its deficiency prevents the decline of neurogenesis in SVZ. No change of neurogenesis is however observed in the p16Ink4a-null dentate gyrus. Here, we hypothesized that p16Ink4a plays a role as a regulator of the self-renewal of the stem cell pool also in the dentate gyrus, and to test this possibility we stimulated the dentate gyrus neural cells of p16Ink4a-null aging mice with physical exercise, a powerful neurogenic activator. We observed that running highly induced the generation of new stem cells in the p16Ink4a-null dentate gyrus, forcing them to exit from quiescence. Stem cells, notably, are not induced to proliferate by running in wild-type (WT) mice. Moreover, p16Ink4a-null progenitor cells were increased by running significantly above the number observed in WT mice. The new stem and progenitor cells generated new neurons, and continued to actively proliferate in p16Ink4a-null mice longer than in the WT after cessation of exercise. Thus, p16Ink4a prevents aging dentate gyrus stem cells from being activated by exercise. Therefore, p16Ink4a may play a role in the maintenance of dentate gyrus stem cells after stimulus, by keeping a reserve of their self-renewal capacity during aging.

## Introduction

The self-renewal ability of stem cells throughout life is a critical issue in cell biology, with implications on development, aging, cancer and neurodegeneration. Neural, as well as muscle and hematopoietic stem cells, self-renew widely, but this potential declines with age (Kuhn et al., 1996; Morrison et al., 1996; Kruger et al., 2002; Conboy et al., 2003; Enwere et al., 2004; Maslov et al., 2004; Molofsky et al., 2006).

The adult brain contains two areas which continue to generate new stem cells and neurons during adulthood, the subgranular zone of the dentate gyrus of the hippocampus and the subventricular zone (SVZ) adjacent to lateral ventricles (Imayoshi et al., 2009).

The generation of new neurons in these two areas occurs from radial glia-like stem cells (Doetsch et al., 1999; Seri et al., 2001). In the dentate gyrus, stem cells (termed type-1) are positive for glial fibrillary acidic protein (GFAP), nestin and Sox2 (Filippov et al., 2003; Kronenberg et al., 2003; Komitova and Eriksson, 2004), while in the SVZ stem cells are called B cells and are positive for GFAP (Doetsch et al., 1999). Stem cells of the dentate gyrus mature into proliferating progenitor cells, classified as type-2a (GFAP-negative, nestin-positive and Sox2-positive), type-2b [positive for nestin and for doublecortin (DCX)] or type-3 (DCX-positive and nestin-negative; Filippov et al., 2003; Fukuda et al., 2003; Kronenberg et al., 2003; Steiner et al., 2006). Progenitor cells mature then into early post-mitotic cells (stage 5)—transiently expressing the Ca-binding protein Calretinin—and into terminally differentiated neurons (stage 6), positive for the late differentiation marker NeuN and for Calbindin (Brandt et al., 2003; Steiner et al., 2004).

In the SVZ the neurogenesis and the self-renewal capacity, as judged from the number of stem cells *in vivo* and the ability to generate neurospheres *in vitro*, respectively, are reduced with age (Enwere et al., 2004; Maslov et al., 2004; Molofsky et al., 2006).

Also in the aged dentate gyrus the production of new neurons from stem cells (type-1 cells) in the subgranular zone decreases (Kuhn et al., 1996; Bizon and Gallagher, 2003; Bondolfi et al., 2004; Couillard-Despres et al., 2009). This is associated to a reduction of hippocampus-dependent memory tasks (van Praag et al., 2005).

A critical role in aging is played by p16Ink4a (Molofsky et al., 2006), which arrests the cell cycle in G1 phase by preventing the association of CDK4 and CDK6 to D-type cyclins (Serrano et al., 1993; Hannon and Beach, 1994), and is involved in the process of senescence (Rayess et al., 2012).

The expression of p16Ink4a becomes detectable only at 1 year of age in many tissues of rodents, including the neurogenic niches, and further increases at 2 years (Zindy et al., 1997; Krishnamurthy et al., 2004; Molofsky et al., 2005, 2006). Also in human tissues, p16Ink4a levels are low until aging takes place (Chkhotua et al., 2003; Ressler et al., 2006).

Indeed, the knockout (KO) of the antiproliferative gene p16Ink4a is sufficient to revert the age-dependent loss of self-renewal and neurospheres generation in the aging SVZ, while no influence is observed in the adult SVZ (in 2-month-old mice), indicating that p16Ink4a contributes to the decline of the self-renewal capacity during aging (Molofsky et al., 2006).

Remarkably, however, the reactivation of self-renewal observed in aging SVZ p16Ink4a KO cells is not seen in the aging dentate gyrus, where p16Ink4a deficiency did not affect the proliferation of progenitor cells or the number of newly generated BrdU^+^NeuN^+^ neurons (Molofsky et al., 2006). Therefore, the role played by p16Ink4a is variable depending on the neural stem cell type.

Some studies showed that, after ablation of the cell cycle inhibitors p21 or Btg1, stem and progenitor cells of the dentate gyrus progressively lose, in an age-dependent fashion, their capability to proliferate and to self-renew (Kippin et al., 2005; Farioli-Vecchioli et al., 2012b). We have also shown that a neurogenic stimulus, such as running or antidepressant fluoxetine, can reactivate the defective self-renewal of Btg1 KO stem cells (Farioli-Vecchioli et al., 2014; Mastrorilli et al., 2017; Micheli et al., 2018a). Notably, the activation of Btg1 KO stem cells observed after a neurogenic stimulus, led us to hypothesize that neural stem cells of adult neurogenic niches during aging or throughout life are endowed with a reserve of proliferative capacity (Farioli-Vecchioli et al., 2014; Mastrorilli et al., 2017; Micheli et al., 2018a), rather than being depleted (Encinas et al., 2011; see Kempermann, 2011 for review about the self-renewal of dentate gyrus stem cells).

Thus, considering the antisenescence and proneurogenic effects of p16Ink4a removal, observed by Molofsky et al. (2006) in the aged SVZ, in this report we sought to test whether a neurogenic stimulus applied to p16Ink4a-null mice could reveal if this gene is another regulator of the reactivation potential for stem cell self-renewal in the dentate gyrus, even though no effect on basal neurogenesis was observed by these authors in the aged dentate gyrus after deletion of p16Ink4a.

More specifically, we asked whether, after removal of p16Ink4a, physical exercise could reactivate aged dentate gyrus stem cells which have reduced self-renewal capacity.

Notably, voluntary physical exercise (running) and fluoxetine are stimuli exerting a powerful neurogenic effect on hippocampal adult progenitor cells (Ryan and Kelly, 2016), and to some extent on SVZ neuroblasts (Bednarczyk et al., 2009; Siopi et al., 2016), but neither of them is able to activate wild-type (WT) stem cells in the dentate gyrus (Kronenberg et al., 2003; Encinas et al., 2006; Steiner et al., 2008; Brandt et al., 2010; Micheli et al., 2017) or in the SVZ (Nasrallah et al., 2010; Ohira and Miyakawa, 2011), with the exception of SVZ stem cells of aged mice after running (Blackmore et al., 2009). However, running is also able to revert the decrease of neuron generation that occurs during aging (van Praag et al., 2005; Marlatt et al., 2012; Siette et al., 2013).

We selected for our analyses 1-year-old p16Ink4a KO mice, because this age is associated with reduced self-renewal and corresponds to the earliest age for a well detectable expression of p16Ink4a; older mice may not perform the physical exercise uniformly and may also develop tumors common to the p16Ink4a KO condition (Sharpless et al., 2001).

We observed that the number of stem cells (type-1) and early progenitor cells (type-2a) in the dentate gyrus of 1-year-old p16Ink4a KO mice was greatly increased by running, while no increase was seen in stem cells of WT mice. The newly generated stem and progenitor cells matured into neurons, indicating that the newly generated stem cells were able to expand and were not limited to self-renewal only. Moreover, the increased generation of new neurons continued in p16Ink4a-null dentate gyrus even for 1 month after cessation of the neurogenic stimulus of running, indicating that the reactivation of stem cells was long-lasting.

## Materials and Methods

### Mouse Lines, Genotyping and Husbandry

The p16Ink4a KO mouse line had been previously generated (Sharpless et al., 2001) and was obtained from the Frederick National Laboratory for Cancer Research (strain number 01XE4; Frederick, MD, USA) as homozygous null mice in FVB background. This strain carries a null allele of p16Ink4a gene, while retaining normal p19Arf function. p16Ink4a KO and p16Ink4a WT strains in C57BL/6 background were then generated by crossing p16Ink4a^+/–^ mice with C57BL/6 strain for at least six generations, until an isogenic progeny was obtained (also referred to as KO and WT throughout the article). The C57BL/6 mouse strain was selected as being the most used for studies of the neurogenic process (Molofsky et al., 2006; Kim et al., 2017) and in order to allow comparison of our results with previous data by Molofsky et al. (2006).

We routinely performed genotyping by PCR analysis, using genomic DNA extracted from tail tips as previously described (Farioli-Vecchioli et al., 2012a, b). The primers used for genotyping were: C015: 5′-ggcaaatagcgccacctat-3′; C016: 5′-gactccatgctgctccagat-3′; C017: 5′-gccgctggacctaataacttc-3′. The primer combination C015/C016 produces DNA fragments of 189 bp in WT mice, while the combination C017/C016 generates fragments of 243 bp in KO mice.

Mice were maintained under standard specific-pathogen-free conditions and were housed in standard cages until they reached 1 year of age. Then, mice were randomly assigned to running wheel or standard cages for 12 days. Run distances were recorded daily with an automatic counter. After 12 days, mice were euthanized or returned to standard cages to be sacrificed at different times after the end of the run, as indicated. The average running wheel distance over the whole experiment (12 days) was 3.51 km/day ± 0.53 (SEM) for WT mice and 3.69 km/day ± 0.66 (SEM) for KO mice, without significant differences (*p* = 0.82 Student’s *t*-test); the total distances run were on average 41.28 km ± 6.67 (SEM) for WT and 41.39 km ± 8.20 (SEM) for KO mice (*p* = 0.99, n WT mice = 16, n KO mice = 13, Student’s *t*-test).

This study was carried out in accordance with the recommendations with the current European Ethical Committee guidelines (directive 2010/63/EU) and the protocol of the Italian Ministry of Health (authorization 1209-2015-PR).

### Dentate Gyrus Dissection, RNA Extraction, Real Time RT-PCR

Two-month-old and 12-month-old p16Ink4a WT and KO mice (three animals per group) were sacrificed. Bilateral dentate gyrus was dissected according to the protocol described by Hagihara et al. (2009) and immediately homogenized in Trizol Reagent (Invitrogen, San Diego, CA, USA). Total cellular RNA extraction and retrotranscription were performed as described previously (Farioli-Vecchioli et al., 2012a). Real-time PCR was carried out with a 7900HT System (Applied Biosystems, Foster City, CA, USA) using SYBR Green I dye chemistry in duplicate samples. Relative quantification was performed by the comparative cycle threshold method (Livak and Schmittgen, 2001). The mRNA expression values were normalized to endogenous control TATA-binding protein gene. Statistical analysis of mRNA expression values was performed by Student’s *t*-test on RQ values. Specific Real time RT-PCR primers were from published murine cDNA sequences. The sequences were: TBP-F: 5′-CCAATGACTCCTATGACCCCTA-3′ and TBP-R: 5′-CAGCCAAGATTCACGGTAGAT-3′; p16Ink4a-F: 5′-GAAGCCGGGGTTTCGC-3′ and p16Ink4a-R: 5′-GCAGAAGAGCTGCTACGTGA-3′; p16Ink4a-F is included within the region deleted in the p16Ink4 KO construct (Sharpless et al., 2001).

### Experimental Design, BrdU Treatment of Mice and Sample Preparation for Immunohistochemistry

**Figure 1:**
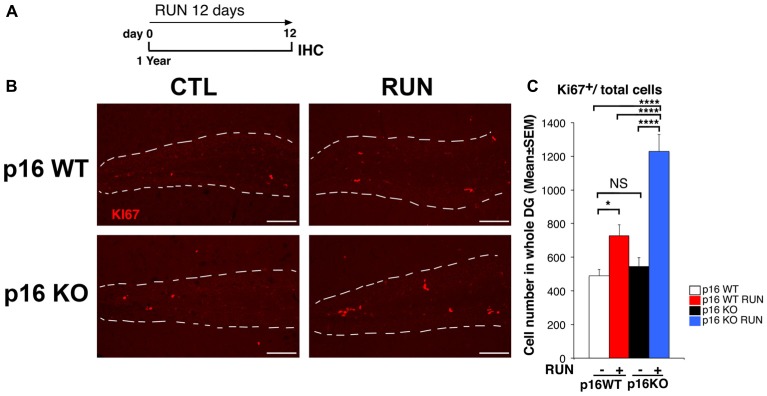
Voluntary running promotes the proliferation of aged dentate gyrus p16Ink4a knockout (KO) cells more than wild-type (WT) cells. **(A)** Experimental timeline: 1-year-old mice, either p16Ink4a WT or KO, were allowed access to running wheels for 12 days, followed by immunohistochemistry analysis. **(B)** Representative images by confocal microscopy showing an increase of cycling progenitor cells (Ki67^+^) in the dentate gyrus of p16Ink4a WT and KO mice subjected to voluntary running exercise, compared to control (sedentary) mice. The white dotted line labels the outer boundaries of the dentate gyrus. Scale bar, 100 μm. **(C)** Number of dividing cells (Ki67^+^) in p16Ink4a WT and KO dentate gyrus. The proliferative effect of running is more marked in p16Ink4a KO dentate gyrus progenitor cells (Two-way ANOVA, running effect, *F*_(1,165)_ = 48.14, *p* < 0.0001; genotype effect *F*_(1,165)_ = 17.563, *p* < 0.0001, followed by analysis of simple effects: *p < 0.05, ****p < 0.0001 or NS *p* > 0.05, Fisher’s PLSD ANOVA *post hoc* test). The numbers of dentate gyrus cells are means ± SEM; four animals per group were analyzed.

**Figure 2:**
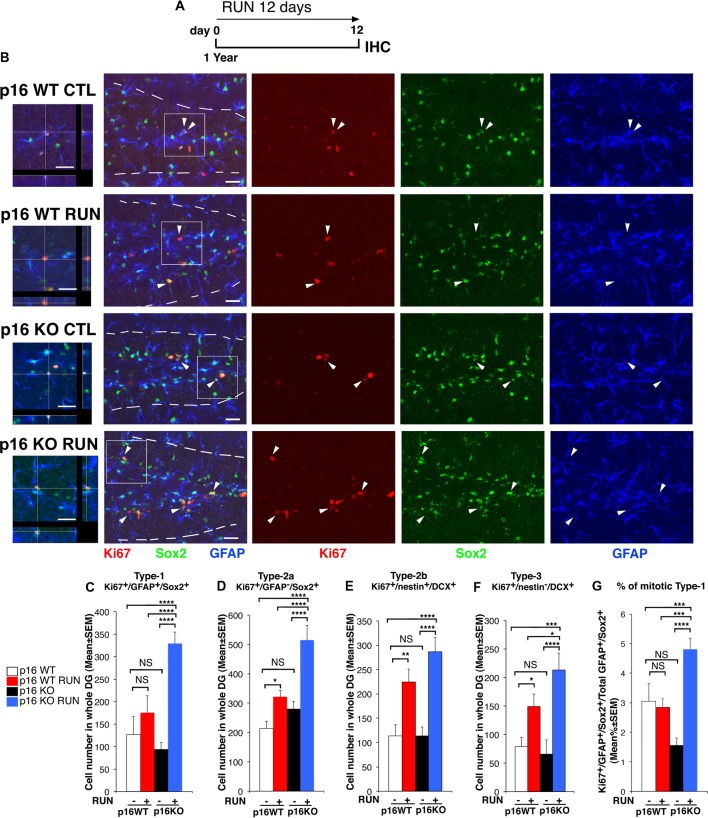
Voluntary running highly stimulates the proliferation of p16Ink4a KO stem cells of the aged dentate gyrus by triggering their entry into the cycle. **(A)** Experimental timeline: 1-year-old mice, either p16Ink4a WT or KO, were allowed voluntary running for 12 days, followed by immunohistochemistry analysis. **(B)** Representative images by confocal microscopy showing that p16Ink4a KO stem cells (Ki67^+^/GFAP^+^/Sox2^+^) are increased by running to an extent higher than in all other conditions. The white dotted line labels the outer boundaries of the dentate gyrus. Arrow heads indicate triple labeled stem cells (Ki67^+^/GFAP^+^/Sox2^+^, in red/blue/green). On the left are represented 3D reconstructions from Z-stack and orthogonal projections of the triple positive cells indicated in the white box (1.25×). Scale bar, 25 μm. **(C)** The number of WT stem cells (type-1, Ki67^+^/GFAP^+^/Sox2^+^) is not affected by running, while **(D)** type-2a progenitor cells (Ki67^+^/GFAP^−^/Sox2^+^) are increased; moreover, p16Ink4a KO type-1 and type-2a cells are significantly augmented by running, relative to all other conditions (two-way ANOVA, running effect: type-1, *F*_(1,165)_ = 46.7, *p* < 0.0001; type-2a, *F*_(1,165)_ = 19.6, *p* < 0.0001). **(E)** The number of type-2b and **(F)** type-3 progenitor cells (Ki67^+^/nestin^+^/DCX^+^ and Ki67^+^/nestin^−^/DCX^+^, respectively) was significantly increased by running in both WT and p16Ink4a KO dentate gyrus (two-way ANOVA, running effect: type-2b, *F*_(1,167)_ = 26.15, *p* < 0.0001; type-3, *F*_(1,167)_ = 18.8, *p* < 0.0001, followed by analysis of simple effects: *p < 0.05, **p < 0.01, ***p < 0.001, ****p < 0.0001 or NS *p* > 0.05, Fisher’s PLSD ANOVA *post hoc* test). **(C–F)** The numbers of dentate gyrus cells are means ± SEM; four animals per group were analyzed. **(G)** The stem cells recruited to the cell cycle, measured as percentage ratio of Ki67^+^/GFAP^+^/Sox2^+^ cells to the total GFAP^+^/Sox2^+^ cells, are significantly increased by running in p16Ink4a KO dentate gyrus above all other conditions [Kruskall-Wallis (d.f. 3) *H* = 43.586, *p* < 0.0001, followed by analysis of simple effects: KO RUN vs. all other groups, *p* ≤ 0.0002, Mann-Whitney *U* test]. Cell ratios are means% ± SEM and were obtained by analyzing the sections of the four animals per group used in **(C)**. Analysis of simple effects: ***p < 0.001, or ****p < 0.0001, Mann-Whitney *U* test.

To examine stem and progenitor cell populations, 1-year-old p16Ink4a WT and KO mice were exposed to running wheels for 12 days and sacrificed 0, 16, and 28 days after the end of the exercise session together with sedentary mice (Figures 1A, 2A, 4A).

**Figure 3:**
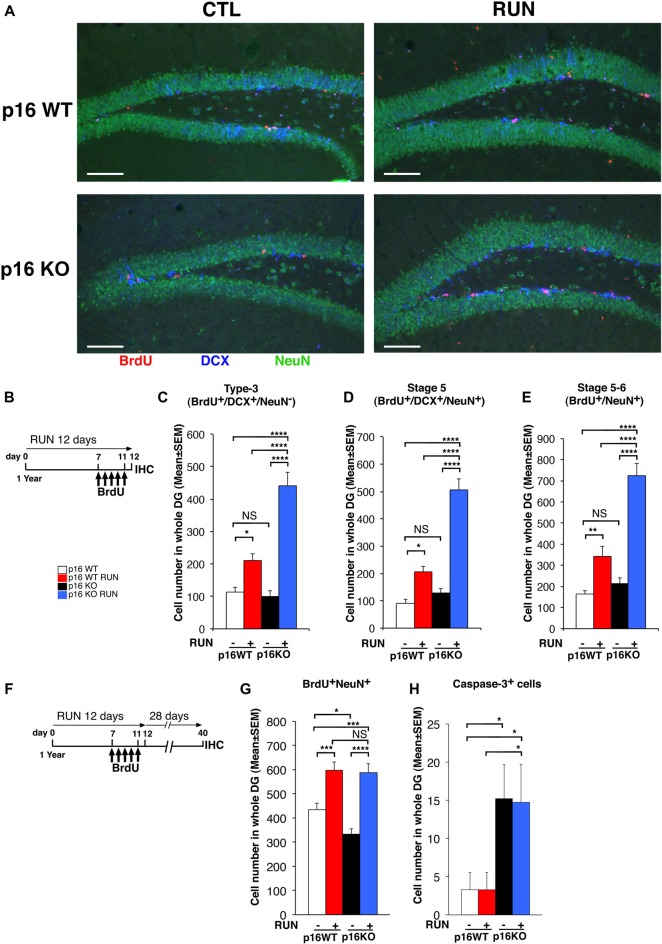
Voluntary running promotes the generation of a greater number of 1- to 5-day-old neurons in the p16Ink4a KO aged dentate gyrus than in WT. **(A)** Representative images by confocal microscopy showing that voluntary running induces a greater production of 1- to 5-day-old neuroblasts (BrdU^+^/DCX^+^/NeuN^−^) and neurons (BrdU^+^/DCX^±^/NeuN^+^) in p16Ink4a KO mice, relative to WT. Scale bar, 100 μm. **(B)** Experimental diagram of exercise and BrdU treatment: 1-year-old mice, either p16Ink4a WT or KO, were allowed voluntary running for 12 days; mice received five daily injections of BrdU (95 mg/kg) during the last days of exercise (from day 7 to day 11), then were subjected to immunohistochemistry analysis. **(C)** The number of 1- to 5-day-old type-3 progenitor cells (BrdU^+^/DCX^+^/NeuN^−^), **(D)** of stage 5 neurons (BrdU^+^/DCX^+^/NeuN^+^), as well as **(E)** stage 5–6 neurons (BrdU^+^/NeuN^+^), was augmented by running in the dentate gyrus of p16Ink4a KO mice above the number of WT mice, either sedentary or running (two-way ANOVA, running effect: type-3, *F*_(1,235)_ = 49.9, *p* < 0.0001; stage 5, *F*_(1,235)_ = 48.7, *p* < 0.0001; stage 5–6, *F*_(1,235)_ = 59.08, *p* < 0.0001, followed by analysis of simple effects: *p < 0.05, **p < 0.01, ****p < 0.0001 or NS *p* > 0.05, Fisher’s PLSD ANOVA *post hoc* test). The numbers of dentate gyrus cells are means ± SEM; five animals per group were analyzed. **(F,G)** Voluntary running stimulates the generation of 28-day-old neurons to the same extent in p16Ink4a KO and in WT dentate gyrus. **(F)** Experimental diagram of exercise and BrdU treatment: 1-year-old mice, either p16Ink4a WT or KO, were allowed voluntary running for 12 days, receiving five daily injections of BrdU (95 mg/kg) in the last days of exercise, followed by 28 days without exercise and then by immunohistochemistry analysis. **(G)** The number of the 28-day-old neurons (BrdU^+^/NeuN^+^) was significantly increased by running to similar values in p16Ink4a WT and KO dentate gyrus (two-way ANOVA, running effect: *F*_(1,206)_ = 45.58, *p* < 0.0001, followed by analysis of simple effects: *p < 0.05, ***p < 0.001, or ****p < 0.0001, Fisher’s PLSD ANOVA *post hoc* test). The numbers of dentate gyrus cells are means ± SEM; five animals per group were analyzed. **(H)** Quantification of the total number of neurons positive for activated Caspase-3 in the same 1-year-old mice used for the quantification shown in graph **(G)**, either p16Ink4a WT or KO. Caspase-3^+^ cells increased significantly in KO dentate gyrus [Kruskall-Wallis (d.f. 3) *H* = 9.49, *p* = 0.023, followed by analysis of simple effects: *p < 0.05, Mann-Whitney *U* test]. The numbers of dentate gyrus cells are means ± SEM; five animals per group were analyzed.

**Figure 4:**
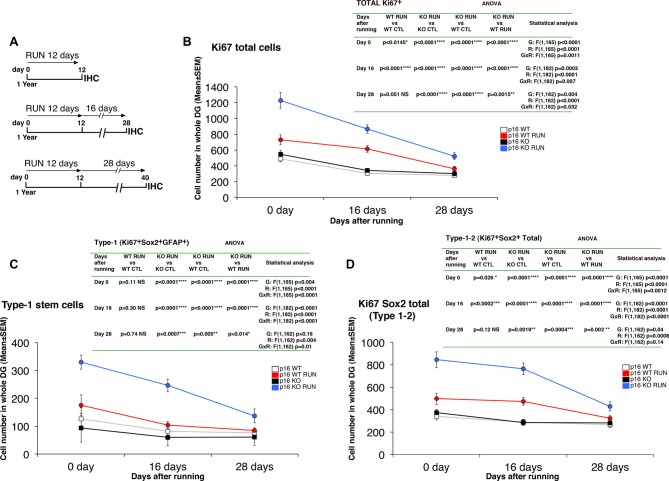
Running triggers a long-term proliferative activation of stem and progenitor cells in the p16Ink4a-null aged mice. **(A)** Experimental timeline of exercise: 1-year-old mice were allowed access to running wheels for 12 days and sacrificed after 0, 16, and 28 days. **(B–D)** Quantification of cycling Ki67^+^ total cells **(B)**, of proliferating stem cells (type-1, Ki67^+^/GFAP^+^/Sox2^+^; **C**), and total Ki67^+^/Sox2^+^ cells (type-1–2; **D**), at the different time points analyzed. Statistical analysis indicates ANOVA *F* test for genotype effect (G), running effect (R), and genotype x running effect (G x R) followed by analysis of simple effects by Fisher’s PLSD ANOVA *post hoc* test. The day 0 time point of Ki67-positive cells and of type-1 cells corresponds to the values shown in Figures 1C, 2C, respectively. Cell number in the dentate gyrus is means ± SEM of the analysis of four animals per group.

In 1-year-old p16Ink4a WT and KO mice, 1- to 5-day-old progenitor cells and neurons were detected by bromodeoxyuridine (BrdU) incorporation, after treatment with five daily injections of BrdU (95 mg/kg i.p.; Sigma Aldrich, St Louis, MO, USA), performed during the last days of running (i.e., running was until day 12, and BrdU injection were from day 7 to day 11; Figure 3B).

The terminally differentiated neurons (28-day-old) were detected 28 days after the end of the 12 days running sessions, following treatment with five daily injections of BrdU (95 mg/kg i.p.) during the last days of exercise (Figure 3F).

Brains were collected after transcardiac perfusion with 4% paraformaldehyde (PFA) in PBS and kept overnight in PFA. Brains were then equilibrated in 30% sucrose and cryopreserved at −80°C.

### Immunohistochemistry (BrdU Labeling, Immunofluorescence)

Immunohistochemistry was performed on serial free-floating sections cut coronally at 40-μm thickness at −25°C in a cryostat from brains embedded in Tissue-Tek OCT (Sakura, Torrence, CA, USA). Sections were then processed immunohistochemically for multiple labeling with BrdU and other cellular markers using fluorescent methods.

Sections were previously permeabilized with 0.3% TritonX-100 in PBS, and then incubated with primary antibodies with 3% normal donkey serum in PBS for 16–18 h.

BrdU incorporation was detected by denaturing DNA through pretreatment of sections with 2N HCl 45 min at 37°C, followed by 0.1 M sodium borate buffer pH 8.5 for 10 min. Sections were then incubated overnight at 4°C with a rat monoclonal antibody against BrdU (AbD Serotech, Raleigh, NC, USA; MCA2060; 1:400).

Cell proliferation was detected also by means of the rabbit monoclonal anti-Ki67 (LabVision Corporation, Fremont, CA, USA; clone SP6; 1:200). The antibodies against Ki67 and BrdU were incubated together with other primary antibodies, so as to visualize the specific subpopulations of dentate gyrus stem, progenitor cells and neurons: mouse monoclonal antibodies against GFAP (Sigma Aldrich; G6171; 1:200) or against nestin (Millipore, Temecula, CA, USA; MAB353; 1:150) or NeuN (Millipore; MAB377; 1:400); goat polyclonal antibodies against Sox2 (Santa Cruz Biotechnology, Santa Cruz, CA, USA; SC-17320; 1:300) or doublecortin (DCX; Santa Cruz Biotechnology; SC-8066; 1:300), or rabbit polyclonal antibody against cleaved (activated) Caspase-3 (Cell Signaling Technology, Danvers, MA, USA; 9661; 1:100).

In order to visualize the antigen, we used secondary antibodies that were all obtained from Jackson ImmunoResearch (West Grove, PA, USA), as follows: a donkey anti-rabbit antiserum Cy3-conjugated (Ki67), a donkey anti-rat antiserum TRITC-conjugated (BrdU), a donkey anti-goat conjugated to Alexa-488 (Sox2) or to Alexa-647 (DCX), a donkey anti-mouse conjugated to Alexa-488 (nestin, NeuN) or to Alexa-647 (NeuN, GFAP), or a donkey anti-rabbit conjugated to Cy3 (Caspase-3).

Nuclei were counterstained by Hoechst 33258 (Sigma Aldrich; 1 mg/ml in PBS).

Confocal Z-stacks with orthogonal projections and single plane images of the immunostained sections were obtained using a TCS SP5 confocal laser scanning microscope (Leica Microsystems, Wetzlar, Germany). Analyses were performed in sequential scanning mode to rule out cross-bleeding between channels.

### Quantification of Cell Numbers

The number of cells positive for each marker was obtained by stereological analysis performed throughout the whole rostro-caudal extent of the dentate gyrus in one-in-six series of 40-μm free-floating coronal sections (240 μm apart), which were analyzed by confocal microscopy. The total estimated number of cells within the dentate gyrus, positive for each of the indicated markers, was obtained by multiplying the average number of positive cells per section by the total number of 40-μm sections including the entire dentate gyrus (about 50–60 sections), as described (Gould et al., 1999; Jessberger et al., 2005; Kee et al., 2007; Farioli-Vecchioli et al., 2008, 2012b). Therefore, about 10 sections per mouse and at least four animals per group were analyzed.

In Figure 2G cell numbers were calculated as percentage ratios of Ki67-type-1 stem cells to the total number of type-1 cells.

Cell number analyses were performed manually by trained experimenters using the I.A.S. software to register positive cells (Delta Sistemi, Rome, Italy).

### Statistical Analysis

The effect of running in p16Ink4a WT and KO mice was statistically analyzed in all experiments using two-way ANOVA, i.e., to test the main effects of genotype or running on the cell number of each cell population. Individual between-group comparisons to test simple effects were carried out by Fisher’s PLSD ANOVA *post hoc* test. In experiments where the data were calculated as percentage of mitotic (proliferating) stem cells (type-1; Figure 2G) or where the labeled cells within each dentate gyrus section were in low number (Caspase-3^+^ cells; Figure 3H), we used—after verifying with the Levene’s test that the equality of variances was not satisfied—non-parametric tests, namely, the Kruskall-Wallis test to analyze the main effects and the Mann-Whitney *U* test to analyze the simple effects.

ANOVA and non-parametric analyses are summarized in Supplementary Tables S1, S2, respectively. The ANOVA data of Figure 4 are directly indicated in figure and therefore are not reported in Supplementary Table S1. Student’s *t*-test was used to compare p16Ink4a mRNA expression data between 2-month-old and 1-year-old WT mice.

These analyses were performed using the StatView 5.0 software (SAS Institute, Cary, NC, USA) and XLSTAT (Addinsoft, Paris, France). Differences were considered statistically significant at *p* < 0.05. All data were expressed as mean values ± SEM.

We defined the minimum sample number needed for each experimental group by *a priori* Power analysis with the G*Power software (Heinrich Heine, Düsseldorf University, Germany).

## Results

Since aging induces a progressive loss of stem/progenitor cell numbers and self-renewal ability in neurogenic niches, our aim was to define whether the deletion of p16Ink4a could allow the reactivation, in response to the neurogenic stimulus of running, of the proliferative potential of aging stem and progenitor cells of the dentate gyrus.

### In Response to Exercise p16Ink4a Knockout Aged Dentate Gyrus Cells Proliferate More Than Wild-Type Cells

We checked and confirmed that p16Ink4a mRNA begins to be well detectable in the dentate gyrus of 1-year-old mice, with levels at the threshold of detectability in 2-month-old mice (Supplementary Figure S1).

Therefore, we submitted aged p16Ink4a WT and KO mice (1-year-old) to voluntary running for 12 days (called here as WT RUN and KO RUN, respectively), by a protocol previously used (Farioli-Vecchioli et al., 2014; Figure 1A). First, we verified that no significant differences were detected between the two genotypes in the running ability (see “Materials and Methods” section, “Mouse cell lines, genotyping and husbandry” Section). At the end of the 12-day exercise schedule we measured the proliferating stem and progenitor cells of the dentate gyrus, identified by Ki67 labeling as cycling cells.

The number of Ki67^+^ cells in the dentate gyrus did not differ, in sedentary mice (CTL), between the p16Ink4a WT and KO genotypes (KO CTL vs. WT CTL, *p* = 0.55; Fisher’s PLSD ANOVA *post hoc* test; Figures 1B,C), in agreement with previous data (Molofsky et al., 2006). However, we observed that running induced a great increase of the total number of proliferating cells in WT mice, as expected, and also in p16Ink4a KO mice; surprisingly, these latter showed a significant increase not only relative to WT control mice, but also relative to WT mice that underwent the running schedule (WT RUN vs. WT CTL, *p* = 0.014; KO RUN vs. WT CTL, *p* < 0.0001 and 151% increase; KO RUN vs. WT RUN, *p* < 0.0001 and 68% increase; Fisher’s PLSD ANOVA *post hoc* test; Figures 1B,C).

This indicated that the ablation of p16Ink4a enabled stem/progenitor cells of the aged dentate gyrus to attain a proliferative capability greater than WT cells under a neurogenic stimulus.

### The Population of p16Ink4a Knockout Aged Stem Cells Is Stimulated by Running to Enter Into the Cell Cycle

Thus, we sought to define whether a specific cell population was responsible for the increased proliferative capability of p16Ink4a KO neural cells following running, using the above protocol (Figure 2A).

We observed that running did not influence the number of proliferating WT stem cells, i.e., type-1 cells, while it increased early progenitor dentate gyrus cells (type-2a cells), identified as Ki67^+^/GFAP^+^/Sox2^+^ and as Ki67^+^/GFAP^−^/Sox2^+^, respectively (WT RUN vs. WT CTL, *p* = 0.11 for type-1 and *p* = 0.04 for type-2a; Fisher’s PLSD ANOVA *post hoc* test; Figures 2B–D). This agrees with previous data showing that the stem cells of the dentate gyrus are not affected by running (Kronenberg et al., 2003; Steiner et al., 2008; Brandt et al., 2010). In contrast, running did cause a striking increase of p16Ink4a KO stem cells as well as of type-2a progenitor cells, above WT cells not only of sedentary but also of exercised mice (KO RUN vs. WT CTL, *p* < 0.0001 and 159% increase for type-1 and 140% increase for type-2a; KO RUN vs. WT RUN, *p* < 0.0001 and 87% increase for type-1, *p* = 0.0002 with 60% increase for type-2a; Fisher’s PLSD ANOVA *post hoc* test; Figures 2B–D).

Likewise, running greatly increased the number of type-2b and type-3 progenitor cells (Ki67^+^/nestin^+^/DCX^+^ and Ki67^+^/nestin-/DCX^+^, respectively) in the p16Ink4a KO dentate gyrus; furthermore, running increased also in WT dentate gyrus the number of type-2b and type-3 progenitor cells, as expected (KO RUN vs. WT CTL, *p* < 0.0001 for type-2b and *p* = 0.0002 for type-3; WT RUN vs. WT CTL, *p* = 0.004 for type-2b and *p* = 0.048 for type-3; Fisher’s PLSD ANOVA *post hoc* test; Figures 2E,F and Supplementary Figure S2). Of note, the number of these progenitor cells in p16Ink4a KO exercised mice exceeded that of exercised WT mice, with type-3 attaining statistical significance (type-3: KO RUN vs. WT RUN, *p* = 0.041 and 42% increase; Fisher’s PLSD ANOVA *post hoc* test; Figures 2E,F).

Thus, the deletion of p16Ink4a conferred stem cells responsiveness to a neurogenic stimulus to which they are not normally susceptible.

We asked whether the increase of the number of proliferating stem cells elicited by running in the p16Ink4a KO aging dentate gyrus was a consequence of a recruitment in the cycle of quiescent cells, or rather of an increased amplification of already cycling stem cells. Thus, we measured the percentage of type-1 stem cells recruited to the cell cycle, i.e., the ratio of Ki67^+^/GFAP^+^/Sox2^+^ cells to the total GFAP^+^/Sox2^+^ cells. This percentage ratio resulted significantly higher in the running p16Ink4a KO mice compared to all other experimental conditions (KO RUN vs. WT CTL or vs. WT RUN, *p* = 0.0001 or *p* = 0.0002, respectively, and about 60% increase in both comparisons; KO RUN vs. KO CTL, *p* < 0.0001 and 200% increase; Mann Whitney *U* test; Figure 2G).This indicated that, in p16Ink4a KO stem cells, running forces quiescent stem cells to enter the cycle, while this effect does not occur in WT cells.

Moreover, we checked whether the total number of quiescent stem cells (Ki67^−^/GFAP^+^/Sox2^+^ cells) was reduced by the increased exit from quiescence induced by exercise. The number of total quiescent cells was somewhat increased by running in both p16Ink4a WT and KO dentate gyrus, relative to the corresponding controls, however without attaining full statistical significance (WT CTL: 5196 ± 385; WT RUN: 6070 ± 319; KO CTL: 6316 ± 363; KO RUN: 6734 ± 315; two-way ANOVA, running effect: *F*_(1,165)_ = 3.4, *p* = 0.06, see full ANOVA data in Supplementary Table S1).

### The Running-Expanded Population of p16Ink4a Knockout Aged Stem Cells Mature Into New Neurons

Next, we sought to define whether the increased generation of stem and progenitor cells elicited by the neurogenic stimulus, observed in the p16Ink4a KO aging dentate gyrus, led to the production of a greater number of neurons. As soon as progenitor cells attain the post-mitotic state, these become early differentiated neurons (stage 5; Kempermann et al., 2004), positive for DCX and for the late neuronal marker NeuN, which then mature in terminally differentiated neurons, DCX-negative and NeuN-positive (Brandt et al., 2003; Steiner et al., 2004).

Thus, we measured the 1- to 5-day-old type-3 progenitor cells and stage 5 early differentiated neurons (detected as BrdU^+^/DCX^+^/NeuN^−^ and BrdU^+^/DCX^+^/NeuN^+^ cells, respectively) at the end of the 12 day exercise period, after five daily injections of BrdU (Figures 3A,B). We observed that both 1- to 5-day-old type-3 progenitor cells and stage 5 neurons were greatly increased by running in the p16Ink4a KO dentate gyrus above the number of WT mice, either sedentary or undergoing exercise (type-3: KO RUN vs. WT CTL, about four-fold increase, *p* < 0.0001; KO RUN vs. WT RUN, about two-fold increase, *p* < 0.0001; stage 5 neurons: KO RUN vs. WT CTL, 5.6-fold increase, *p* < 0.0001; KO RUN vs. WT RUN, 2.4-fold increase, *p* < 0.0001; Fisher’s PLSD ANOVA *post hoc* test; Figures 3A–D). Also, the 1- to 5-day-old stage 5–6 neurons (BrdU^+^NeuN^+^) were similarly increased by running in the p16Ink4a KO dentate gyrus (KO RUN vs. WT CTL, 4.3-fold increase, *p* < 0.0001; KO RUN vs. WT RUN, 2.1-fold increase, *p* < 0.0001; Fisher’s PLSD ANOVA *post hoc* test; Figure 3E).

We then asked whether the great increase above WT of the number of new p16Ink4a KO 1- to 5-day-old neurons, induced by running, remained detectable also after their maturation into terminally differentiated neurons. Therefore, we analyzed the 28-day-old BrdU^+^NeuN^+^ cells, labeled with five daily injections of BrdU carried out during the last days of the running exercise; neurons were then allowed to differentiate during the following 28 days (see scheme, Figure 3F). We observed that the number of 28-day-old neurons was significantly augmented by running both in WT and in p16Ink4a KO dentate gyrus, relative to the number of sedentary controls (WT RUN vs. WT CTL, 37% increase, *p* = 0.0002; KO RUN vs. KO CTL, 76% increase, *p* < 0.0001; Fisher’s PLSD ANOVA *post hoc* test; Figure 3G).

However, WT and p16Ink4a KO dentate gyrus neurons were augmented by running to equivalent numbers (WT RUN vs. KO RUN, *p* = 0.81; Fisher’s PLSD ANOVA *post hoc* test). Therefore, we asked whether the lack of a more pronounced increase of 28-day-old neurons in p16Ink4a KO running mice, compared with WT running mice (Figure 3G), might be consequence of a reduced survival.

Thus, we analyzed the number of apoptotic cells—identified as total cells positive for activated Caspase-3—in the dentate gyrus of the same mice that were submitted to exercise and then left sedentary for the following 28 days (i.e., according to the protocol of Figure 3F).We observed that the p16Ink4a KO dentate gyrus, either running or sedentary, presented a significant increase of apoptotic cells relative to WT (KO RUN or KO CTL vs. WT CTL, *p* = 0.046 and *p* = 0.021, respectively, and about 4.5-fold increase in both comparisons; Mann Whitney *U* test; Figure 3H). This raises the possibility that the increase stimulated by running of the number of 28-day-old neuron, observed in p16Ink4a KO dentate gyrus, did not exceed the number of WT neurons in running mice (as instead observed for 1- to 5-day-old neurons, Figure 3E) because of a reduced survival.

### The New p16Ink4a-Null Aged Stem and Progenitor Cells Show Long-Term Proliferation Activity After Running

An important question raised by the strong proliferation elicited by running on stem and progenitor cells of p16Ink4a KO dentate gyrus was whether this proliferative effect lasted after the end of the neurogenic stimulus or was short-term. To this aim, we performed a time course experiment, where the proliferative state of stem and progenitor cells was analyzed at progressively longer intervals of time after the end of the running stimulus, i.e., 0 days, 16 days, or 28 days after the end of the running sessions (Figure 4A).

We analyzed the number of total cycling cells (Ki67^+^), of proliferating stem cells (type-1, Ki67^+^/GFAP^+^/Sox2^+^), and of proliferating Ki67^+^/Sox2^+^ total cells (i.e., type-1–2; Figures 4B–D). All cell numbers slowly decreased throughout time after cessation of running. However, we observed that in running p16Ink4a KO mice the number of Ki67^+^ total cells, of stem cells and of type-1–2 cells remained significantly above the other groups up to 28 days after the end of the running sessions (Ki67^+^ cells: KO RUN vs. WT CTL or vs. WT RUN at day 28, *p* < 0.0001 and *p* = 0.0015, respectively, Figure 4B; type-1 cells: KO RUN vs. WT CTL or vs. WT RUN at day 28, *p* = 0.005 and *p* = 0.014, respectively, Figure 4C; type-1–2 cells: KO RUN vs. WT CTL or vs. WT RUN at day 28, *p* = 0.0004 and *p* = 0.002, respectively, Figure 4D; Fisher’s PLSD ANOVA *post hoc* test). Conversely, in running WT mice the number of Ki67^+^ total cells and of type-1–2 cells remained significantly above control only until 16 days after the end of the running exercise, whereas after 28 days the number of cells in WT RUN mice did not differ from control WT (Ki67^+^ cells: WT RUN vs. WT CTL at day 28, *p* = 0.051, Figure 4B; type-1–2 cells: WT RUN vs. WT CTL at day 28, *p* = 0.12, Figure 4D; Fisher’s PLSD ANOVA *post hoc* test). Moreover, in running WT mice the number of stem cells never exceeded that of control WT at any time interval analyzed (type-1 cells: WT RUN vs. WT CTL at day 0, *p* = 0.11, or at day 16, *p* = 0.30, or at day 28, *p* = 0.74; Fisher’s PLSD ANOVA *post hoc* test; Figure 4C).

This indicated that the activation of proliferation, induced in p16Ink4a KO stem and progenitor cells by the neurogenic stimulus of running, although slowly decreasing in absolute values, nevertheless persisted significantly higher than in WT dentate gyrus for 28 days after cessation of running.

## Discussion

During aging the ongoing process of generation of new stem/progenitor cells and neurons in the dentate gyrus decreases (Kuhn et al., 1996; Bizon and Gallagher, 2003; van Praag et al., 2005), and this is accompanied by a lower ability to perform hippocampus-dependent learning and memory tasks (Drapeau and Nora Abrous, 2008). This is caused by a decline of the ability of stem/progenitor cells to self-renew, which implies a reduced ability to replace the cells that are lost because of turnover or disease (Apple et al., 2017). However, although the hippocampal proliferating stem/progenitor cells decreases up to four-fold during aging (Jin et al., 2003; Lugert et al., 2010), the density of neural stem/progenitor cells in the subgranular zone of the dentate gyrus remains quite constant, indicating that a quiescent pool of Sox2-positive cells remains open to activation (Lugert et al., 2010). Consistently, a neurogenic stimulus such as physical exercise is able to overcome the age-dependent decay of hippocampal neurogenesis in 9–20-month-old mice, by reactivating neurogenesis of at least 50% of the control level of young animals (van Praag et al., 2005; Marlatt et al., 2012; Siette et al., 2013). Therefore, a quota of neural stem cells during aging does not undergo senescence, the condition of permanent exit from the cell cycle, but can be reactivated.

Although overexpression of p16Ink4a, together with p19Arf (encoded by the same Ink4/Arf locus), does not affect the process of aging (Matheu et al., 2004), p16Ink4a is a marker of aging in several tissues including brain (Zindy et al., 1997; Krishnamurthy et al., 2004) and certainly plays a part as an inducer of senescence (Beauséjour et al., 2003; Rayess et al., 2012), such that it has been proposed that this process may protect the physiological longevity. Indeed, deletion of p16Ink4a induces an increase of neurogenesis in the aging SVZ (i.e., of neurospheres generation), but not in the aging dentate gyrus (Molofsky et al., 2006).

Therefore, we asked whether p16Ink4a may also maintain the quiescence of aging dentate gyrus stem cells, and whether this may represent a blockade against the depletion of the pool of stem cells. We hypothesized that, although the depletion of p16Ink4a is not sufficient in itself to stimulate the proliferation in the dentate gyrus neither of stem nor of progenitor cells, even in aged mice, as observed by Molofsky et al. (2006), nevertheless a preserving action on stem cells by p16Ink4a may be unmasked in KO cells following a neurogenic stimulus, such as that provided by running. In fact, we show that in 1-year-old mice, i.e., at the age when p16Ink4a levels become detectable in neurogenic niches (Molofsky et al., 2006), after a period of voluntary running of 12 days, p16Ink4a KO dentate gyrus stem cells (type-1) are very highly activated, about two-fold above control levels in either sedentary or exercised WT mice. Of note, running has no effect on dentate gyrus stem cells in WT mice, as expected (Kronenberg et al., 2003; Steiner et al., 2008; Brandt et al., 2010). This evidence of activation of dentate gyrus stem cells is similar to that observed after a neurogenic stimulus (running or fluoxetine) in stem cells depleted of the quiescence-maintaining gene Btg1 (Farioli-Vecchioli et al., 2014; Micheli et al., 2018a).

Moreover, the percentage of mitotic stem cells is highly increased by running in p16Ink4a KO dentate gyrus, indicating that KO stem cells are induced by exercise to exit from a state of quiescence and proliferate. Additionally, given that in specific conditions, such as the deletion of p16Ink4a and p53, senescence is removed (Beauséjour et al., 2003), it cannot be excluded that a quota of stem cells in a state of senescence is reactivated by running. In fact, running regulates the expression of a number of factors with neurotrophic activity that may also affect the decision of the stem cell to enter the cell cycle (e.g., BDNF or BMP4; see for review Bolijn and Lucassen, 2015; Micheli et al., 2018b).

Speculating on the molecular mechanisms involved in the observed reentry of stem cells into the cycle, this may depend on the interaction between the pathways through which p16Ink4a negatively controls cell proliferation, and by which running potentiates neurogenesis. Notably, p16Ink4a is one of the Ink4 family genes inhibiting the function not only of cyclin D1, but also of cyclin D2, which in the adult dentate gyrus is required to modulate the maturation of neural stem/progenitor cells (Kowalczyk et al., 2004; see for review Farioli-Vecchioli and Tirone, 2015). The proliferative neurogenic effect of running, on the other hand, requires the expression of serotonin, which impacts on Sox2-positive cells (Klempin et al., 2013). Moreover, running induces BDNF and adiponectin, which have a positive effect on hippocampal cell proliferation (Katoh-Semba et al., 2002; Zhang et al., 2011), whereas running inhibits BMP4, which negatively regulates stem/progenitor cell proliferation (Gobeske et al., 2009). Thus, the activation of p16Ink4a-null aging dentate gyrus stem cells by running is an age-dependent process, plausibly subject to several cell non-autonomous (running) and cell autonomous (p16Ink4a KO) converging mechanisms.

Analyzing the successive steps of stem cell maturation, i.e., the type-2 and type-3 progenitor cells—which proceed from stem cells but, unlike stem cells, are increased by running—we observe that in the p16Ink4a-null aged dentate gyrus they were still highly enhanced by running and in particular type-2a and type-3 cells were found in numbers significantly higher than in exercised WT mice.

Further tracing the steps of maturation of stem cells in our model, we observe that in the p16Ink4a KO aged dentate gyrus they generate early 1- to 5-day-old neurons, which are still in great excess, in comparison with the neurons induced by running in WT mice (more than four-fold increase). We take this data as evidence that the stem cells generated in excess in the p16Ink4a KO dentate gyrus after running fully develop into neurons, after the intermediate maturation step as progenitor cells. Nevertheless, the quantitative analysis performed for 28-day-old neurons shows that these are induced by physical exercise in p16Ink4a KO to the same numbers as in the WT dentate gyrus, rather than in excess. This less pronounced increase of neurogenesis induced by running, existing in p16Ink4a KO 28-day-old neurons compared to 5-day-old neurons, suggests that during their development, a number of neurons die before attaining 28 days of age. In fact, this idea is confirmed by a large increase of apoptosis observed in the dentate gyrus of p16Ink4a KO mice, either exercised or sedentary, analyzed 28 days after running with activated Caspase-3.

This increase of apoptosis is consistent with the notion that the inactivation of molecules that regulate the G1 to S phase transition generates a conflict between ongoing opposing proliferative and antiproliferative stimuli, which leads to cell death, as for instance is shown for the ablation of pRb (Lee et al., 1994). In addition, plausibly, the neurogenic stimulus in the p16Ink4a-null dentate gyrus generates stem cells developing into neurons in such excess that part of them does not survive, possibly for insufficient levels of neurotrophic factors in the surrounding milieu (Yamaguchi and Miura, 2015).

An interesting feature of the over-production of stem and progenitor cells triggered by exercise, is that the effect of the neurogenic stimulus appears to be long-lasting and more protracted in p16Ink4a KO stem/progenitor cells, as these show increased proliferation still 28 days after cessation of the running stimulus, while WT stem and progenitor cells do not. This longer-lasting effect of running in p16Ink4a KO stem/progenitor cells suggests that the increase of neurogenesis is somewhat stabilized, relative to the WT. One possible stabilizing factor might be an acceleration of the cell cycle. In fact, in the dentate gyrus of Btg1 KO mice we also observed a great increase of stem cell numbers, induced by running, above exercised WT mice, in concomitance with an acceleration of their cell cycle, which we suggested to be a factor involved in the increase of neurogenesis (Farioli-Vecchioli et al., 2014; Farioli-Vecchioli and Tirone, 2015). However, the decrease of cell cycle length—although it may have a stabilizing effect on neurogenesis—could be a consequence, rather than the cause of the increased neurogenesis. Future studies will be necessary to define this aspect in the p16Ink4a KO dentate gyrus.

Therefore, our data indicate that dentate gyrus stem cells have a hidden proliferation potential, which is maintained also during aging by p16Ink4a. This kinase inhibitor, therefore, not only controls senescence, but is also a regulator of the self-renewal potential of dentate gyrus neural stem cells during aging.

Overall, this suggests that dentate gyrus neural stem cells undergo a process of self-renewal controlled by a network of selected cell cycle inhibitors, of which we have identified so far p16Ink4a and Btg1 (Farioli-Vecchioli et al., 2014). Such a process is flexible and expandable, rather than rigidly determined, in function of the levels of these regulatory genes, which, in normal conditions as well as after a neurogenic stimulus, act to preserve the depletion of the stem cell pool by negatively controlling their proliferation. The idea of a flexible process of self-renewal of stem cells is consistent with what proposed by Bonaguidi et al. (2011), who suggested that stem cells remain able to self-renew throughout their life, although losing proliferative potential, in contraposition to what other studies propose, i.e., that the stem cell pool is fated to gradually decrease, as stem cells are steadily converted into neurons and astrocytes (Encinas et al., 2011; see Kempermann, 2011, for review).

In fact, our data show that the self-renewal capability of stem cells not only remains potentially active during aging, but that it can be enhanced in appropriate conditions.

It is worth noting, however, that the activation of proliferation in p16Ink4a-null aged dentate gyrus cells after stimulus—although persisting longer than in WT—undergoes a progressive decline, hence the neural cell tends to return towards an homeostatic condition of baseline proliferation. We cannot exclude, however, that after a prolonged period of enhanced expansion in the absence of p16Ink4a the reserve of stem cell self-renewal may undergo exhaustion, in analogy with that observed after deletion of the gene Pten (Bonaguidi et al., 2011). More generally, a shift toward depletion or preservation of the stem cell pool might depend on many variables, such as age, regulatory genes expression, neurogenic niche state, and the type and duration of the neurogenic stimulus.

Further studies will be necessary to identify the complete network of genes involved in the control of stem cell self-renewal. Such knowledge may lead to new strategies aimed to reactivate and expand neural stem cells for therapy of neurodegenerative diseases or traumatic brain injury (see Weston and Sun, 2018, for review). In fact, although there is some negative evidence of adult neurogenesis in humans (Sorrells et al., 2018), this has been detected by some groups (e.g., Boldrini et al., 2018), and a comprehensive model has been forwarded proposing that expansion of progenitor cells takes place, albeit to a lower extent than in mouse, followed by a long postmitotic phase (Kempermann et al., 2018).

## Data Availability

The datasets for this manuscript are not publicly available because in this manuscript were generated data that are not of the type required to be made publicly available (e.g., they are not genomic or expression datasets). In fact we have generated excel files containing the raw data of histological analyses, which are stored in the computers of our laboratory and that of course can be made available to reviewers or researchers if they request them. Moreover, the whole detailed statistical analyses of the raw data is presented in Supplementary Tables S1, S2 and in Figure 4. Requests to access the datasets should be directed to felice.tirone@cnr.it.

## Author Contributions

LM, GD and FT contributed to the conception and design of the study. LM, GD, MC, AF and RS carried out the experimental work. FT performed the statistical analysis and wrote the article.

## Conflict of Interest Statement

The authors declare that the research was conducted in the absence of any commercial or financial relationships that could be construed as a potential conflict of interest.

## Supplementary Material

FIGURE S1 p16Ink4a mRNA expression increases with age in the dentate gyrus. Real-time PCR analysis was performed on RNA obtained from the dentate gyrus of 2- and 12-month-old p16Ink4a wild-type and knockout mice. Three mice per group were analyzed. Average ± SEM values were obtained by analyzing the dentate gyrus of each of the three mice with three technical replicates, and are shown as fold change relative to the control sample (one 2-month-old p16Ink4a wild-type mouse), which was set to unity. TATA-binding protein mRNA was used as endogenous control for normalization. ****p* < 0.001, Student’s *t*-test. ND: Not Detectable.

FIGURE S2 Representative images by confocal microscopy of dividing type-1–2a (Ki67^+^/nestin^+^/DCX^−^), type-2b (Ki67^+^/nestin^+^/DCX^+^) and type-3 cells (Ki67^+^/nestin^−^/DCX^+^) in the p16Ink4a wild-type and knockout dentate gyrus of sedentary and exercised mice. Ki67^+^, nestin^+^, DCX^+^ cells are in green, red, and blue, respectively. Nuclei are identified by Hoechst 33258 (panel on the right, in light blue). The arrowhead indicates a proliferating radial glia-like type-1 cell, while arrows indicate type-2ab progenitor cells, and thin arrows indicate type-3 progenitor cells. The analysis of the number of type-2b and type-3 progenitor cells is shown in Figures 2E,F and in Supplementary Tables S1, S2. Scale bar, 30 μm.

TABLE S1 Two-way ANOVA analyses of experiments (main effects are analyzed by Fisher *F* test followed by analysis of simple effects with PLSD test).

TABLE S2 Levene’s test analysis of normality of variances, followed by non-parametric analysis of main factor effects with Kruskal-Wallis test and simple effect analysis with Mann-Whitney U test.
