# Supplementary figures and images for: p16Ink4a Prevents the Activation of Aged Quiescent Dentate Gyrus Stem Cells by Physical Exercise
Source: Front Cell Neurosci. 2019 Feb 7;13:10. doi: 10.3389/fncel.2019.00010 (PMC6374340; doi:10.3389/fncel.2019.00010)

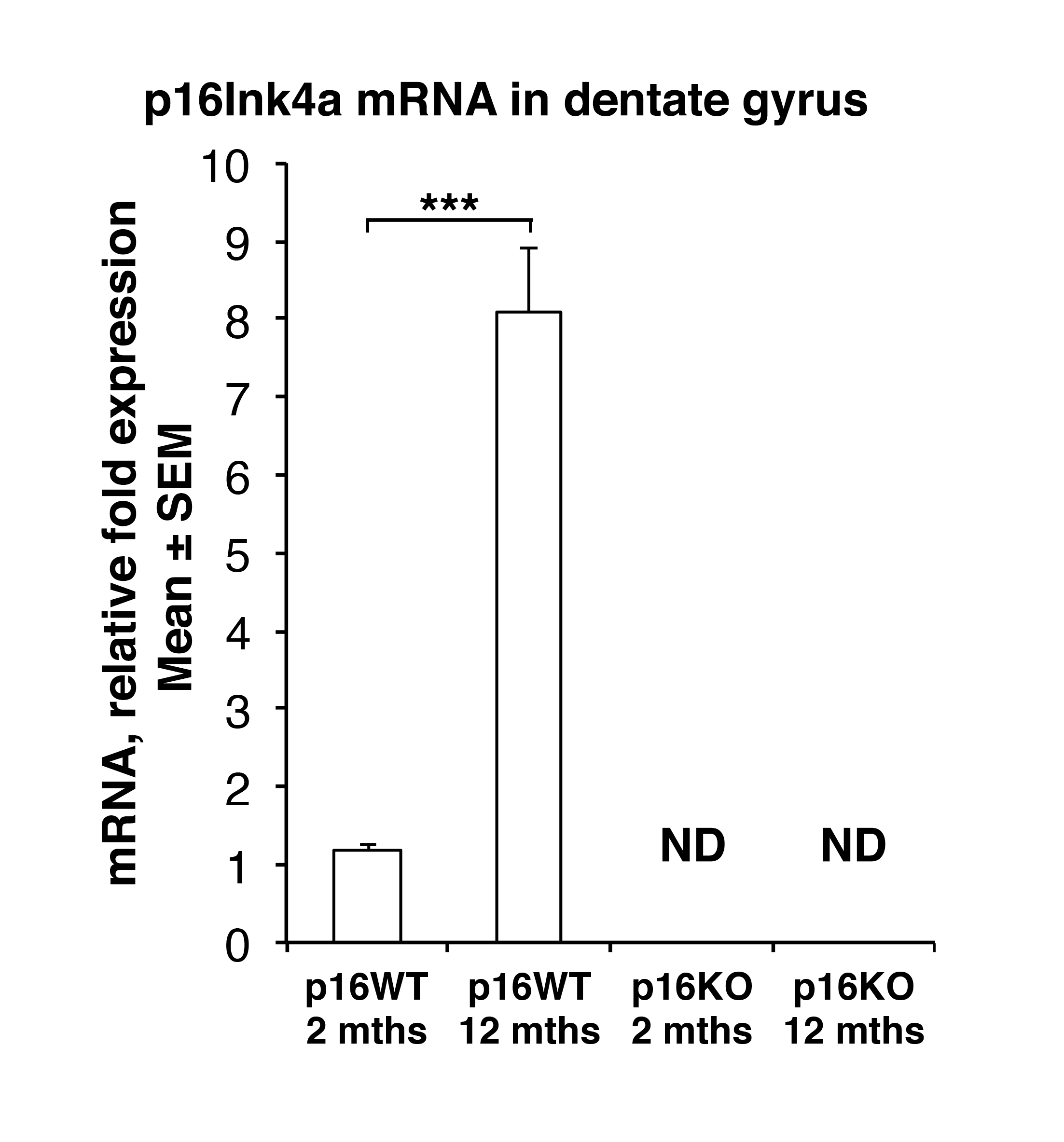

Supplement: FIGURE S1 — p16Ink4a mRNA expression increases with age in the dentate gyrus. Real-time PCR analysis was performed on RNA obtained from the dentate gyrus of 2- and 12-month-old p16Ink4a wild-type and knockout mice. Three mice per group were analyzed. Average ± SEM values were obtained by analyzing the dentate gyrus of each of the three mice with three technical replicates, and are shown as fold change relative to the control sample (one 2-month-old p16Ink4a wild-type mouse), which was set to unity. TATA-binding protein mRNA was used as endogenous control for normalization. ***p < 0.001, Student’s t-test. ND: Not Detectable. [file Image_1.tif]

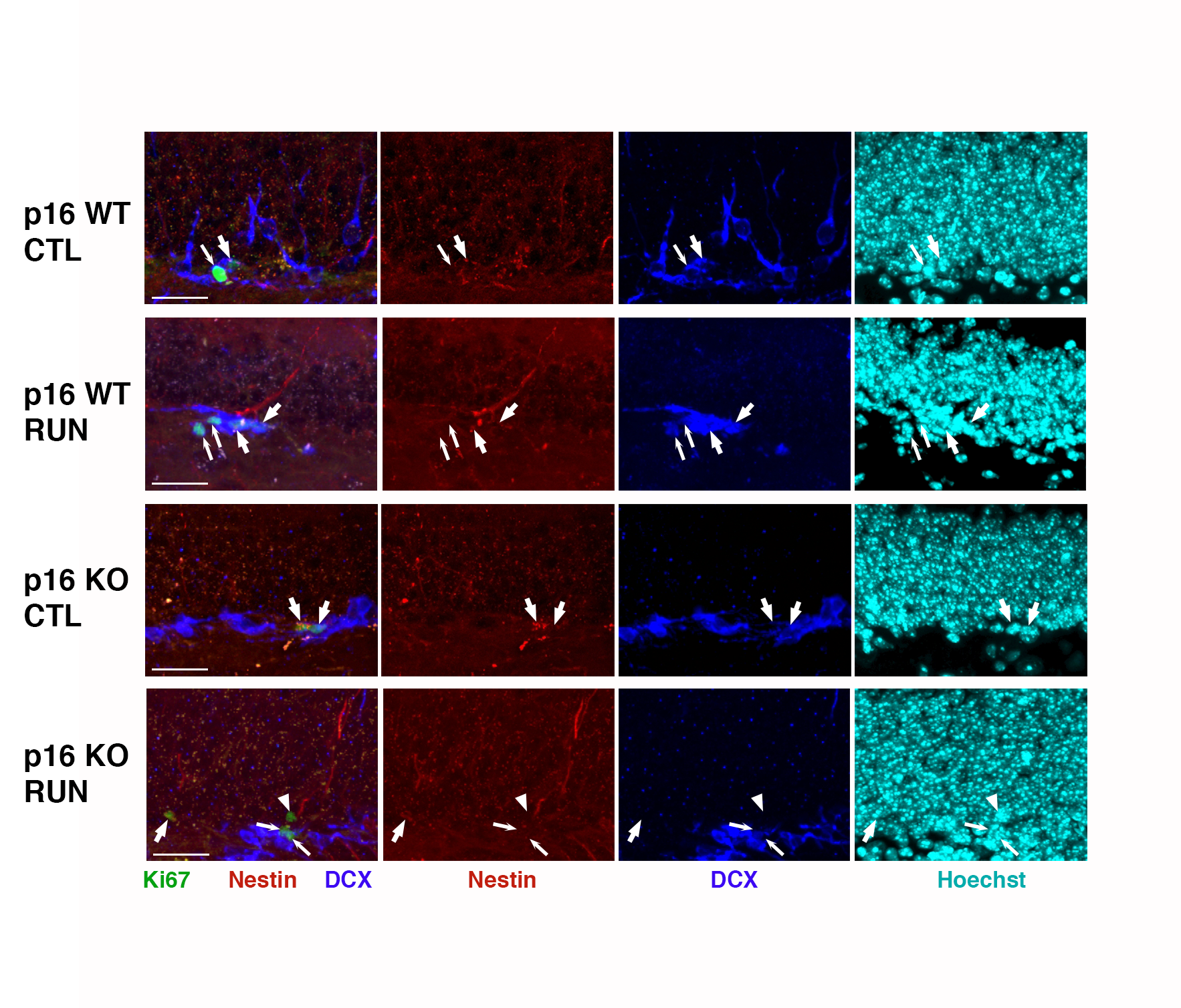

Supplement: FIGURE S2 — Representative images by confocal microscopy of dividing type-1–2a (Ki67+/nestin+/DCX−), type-2b (Ki67+/nestin+/DCX+) and type-3 cells (Ki67+/nestin−/DCX+) in the p16Ink4a wild-type and knockout dentate gyrus of sedentary and exercised mice. Ki67+, nestin+, DCX+ cells are in green, red, and blue, respectively. Nuclei are identified by Hoechst 33258 (panel on the right, in light blue). The arrowhead indicates a proliferating radial glia-like type-1 cell, while arrows indicate type-2ab progenitor cells, and thin arrows indicate type-3 progenitor cells. The analysis of the number of type-2b and type-3 progenitor cells is shown in Figures 2E,F and in Supplementary Tables S1, S2. Scale bar, 30 μm. [file Image_2.tif]
