# Supplementary material for: p16Ink4a Prevents the Activation of Aged Quiescent Dentate Gyrus Stem Cells by Physical Exercise
Source: Front Cell Neurosci. 2019 Feb 7;13:10. doi: 10.3389/fncel.2019.00010 (PMC6374340; doi:10.3389/fncel.2019.00010)
Supplement: TABLE S1 — Two-way ANOVA analyses of experiments (main effects are analyzed by Fisher F test followed by analysis of simple effects with PLSD test). [file Table_1.DOCX]

| Experiment | Two-way ANOVA  factors | Two-way ANOVA  DF | Two-way ANOVA F-Value | Two-way ANOVA  P-Value | Fisher’s PLSD Post-hoc comparisons |
| --- | --- | --- | --- | --- | --- |
| Figure 1C  *Ki67* | Genotype  Treatment  Genotype × Treatment | 1,165  1,165  1,165 | 17.563  48.140  11.109 | <0.0001  <0.0001  0.0011 | p16KO-CTL vs p16KO-RUN <0.0001  p16KO-CTL vs p16WT-CTL 0.5504  p16KO-CTL vs p16WT-RUN 0.0554  p16KO-RUN vs p16WT-CTL <0.0001  p16KO-RUN vs p16WT-RUN <0.0001  p16WT-CTL vs p16WT-RUN 0.0145 |
| Figure 2C  *Type-1*  (ki67 Sox2+GFAP+) | Genotype  Treatment  Genotype × Treatment | 1,165  1,165  1,165 | 8.973  46.691  21.042 | 0.0032  <0.0001  <0.0001 | p16KO-CTL vs p16KO-RUN <0.0001  p16KO-CTL vs p16WT-CTL 0.2773  p16KO-CTL vs p16WT-RUN 0.0071  p16KO-RUN vs p16WT-CTL <0.0001  p16KO-RUN vs p16WT-RUN <0.0001  p16WT-CTL vs p16WT-RUN 0.1139 |
| Figure 2D  *Type-2a*  (ki67 Sox2+ GFAP-) | Genotype  Treatment  Genotype × Treatment | 1,165  1,165  1,165 | 13.717  19.630  3.691 | 0.0003  <0.0001  0.0564 | p16KO-CTL vs p16KO-RUN <0.0001  p16KO-CTL vs p16WT-CTL 0.2136  p16KO-CTL vs p16WT-RUN 0.4318  p16KO-RUN vs p16WT-CTL <0.0001  p16KO-RUN vs p16WT-RUN 0.0002  p16WT-CTL vs p16WT-RUN 0.0476 |
| *Total quiescent stem cells* (data in Results)  (Total Ki67-Sox2+GFAP+) | Genotype  Treatment  Genotype × Treatment | 1,165  1,165  1,165 | 6.527  3.421  0.426 | 0.0115  0.0662  0.5150 | p16KO-CTL vs p16KO-RUN 0.3829  p16KO-CTL vs p16WT-CTL 0.0266  p16KO-CTL vs p16WT-RUN 0.6211  p16KO-RUN vs p16WT-CTL 0.0020  p16KO-RUN vs p16WT-RUN 0.1744  p16WT-CTL vs p16WT-RUN 0.0882 |
| Figure 2E  *Type-2b*  (ki67nestin+ DCX-) | Genotype  Treatment  Genotype × Treatment | 1,167  1,167  1,167 | 1.301  26.516  1.287 | 0.2556  <0.0001  0.2583 | p16KO-CTL vs p16KO-RUN <0.0001  p16KO-CTL vs p16WT-CTL 0.9968  p16KO-CTL vs p16WT-RUN 0.0061  p16KO-RUN vs p16WT-CTL <0.0001  p16KO-RUN vs p16WT-RUN 0.0644  p16WT-CTL vs p16WT-RUN 0.0043 |
| Figure 2F  *Type-3*  (ki67nestin-DCX+) | Genotype  Treatment  Genotype × Treatment | 1,167  1,167  1,167 | 0.987  18.829  2.314 | 0.3220  <0.0001  0.1301 | p16KO-CTL vs p16KO-RUN <0.0001  p16KO-CTL vs p16WT-CTL 0.7393  p16KO-CTL vs p16WT-RUN 0.0243  p16KO-RUN vs p16WT-CTL 0.0002  p16KO-RUN vs p16WT-RUN 0.0412  p16WT-CTL vs p16WT-RUN 0.0489 |
| Figure 3C  *Type-3*  (BrdU+DCX+NeuN-) | Genotype  Treatment  Genotype × Treatment | 1,235  1,235  1,235 | 12.633  49.900  15.333 | 0.0005  <0.0001  0.0001 | p16KO-CTL vs p16KO-RUN <0.0001  p16KO-CTL vs p16WT-CTL 0.7915  p16KO-CTL vs p16WT-RUN 0.0243  p16KO-RUN vs p16WT-CTL <0.0001  p16KO-RUN vs p16WT-RUN <0.0001  p16WT-CTL vs p16WT-RUN 0.0405 |
| Figure 3D  *Stage 5*  (BrdU+DCX+NeuN+) | Genotype  Treatment  Genotype × Treatment | 1,235  1,235  1,235 | 23.320  48.751  13.853 | <0.0001  <0.0001  0.0002 | p16KO-CTL vs p16KO-RUN <0.0001  p16KO-CTL vs p16WT-CTL 0.4185  p16KO-CTL vs p16WT-RUN 0.1685  p16KO-RUN vs p16WT-CTL <0.0001  p16KO-RUN vs p16WT-RUN <0.0001  p16WT-CTL vs p16WT-RUN 0.0340 |
| Figure 3E  *Stage 5-6*  (BrdU+NeuN+) | Genotype  Treatment  Genotype × Treatment | 1,235  1,235  1,235 | 23.706  59.086  13.887 | <0.0001  <0.0001  0.0002 | p16KO-CTL vs p16KO-RUN <0.0001  p16KO-CTL vs p16WT-CTL 0.4028  p16KO-CTL vs p16WT-RUN 0.0701  p16KO-RUN vs p16WT-CTL <0.0001  p16KO-RUN vs p16WT-RUN <0.0001  p16WT-CTL vs p16WT-RUN 0.0101 |
| Figure 3G  *28 day-old neurons*  BrdUNeuN | Genotype  Treatment  Genotype × Treatment | 1,206  1,206  1,206 | 3.22  45.587  2.168 | 0.0742  <0.0001  0.1425 | p16KO-CTL vs p16KO-RUN <0.0001  p16KO-CTL vs p16WT-CTL 0.0238  p16KO-CTL vs p16WT-RUN <0.0001  p16KO-RUN vs p16WT-CTL 0.0005  p16KO-RUN vs p16WT-RUN 0.8175  p16WT-CTL vs p16WT-RUN 0.0002 |

Table S1. Two-way ANOVA analyses of experiments (main effects are analyzed by Fisher F test followed

by analysis of simple effects with PLSD test)
