# Supplementary material for: p16Ink4a Prevents the Activation of Aged Quiescent Dentate Gyrus Stem Cells by Physical Exercise
Source: Front Cell Neurosci. 2019 Feb 7;13:10. doi: 10.3389/fncel.2019.00010 (PMC6374340; doi:10.3389/fncel.2019.00010)
Supplement: TABLE S2 — Levene’s test analysis of normality of variances, followed by non-parametric analysis of main factor effects with Kruskal-Wallis test and simple effect analysis with Mann-Whitney U test. [file Table_2.DOCX]

Table S2. Levene’s test analysis of normality of variances, followed by non-parametric analysis of main factor effects with Kruskal-Wallis test and simple effect analysis with Mann-Whitney U test.

| Experiment | Levene’s test P value | Kruskal-Wallis  DF | Kruskal-Wallis H value | Kruskal-Wallis P value | Mann-Whitney U test Post-hoc comparisons |
| --- | --- | --- | --- | --- | --- |
| Figure 2G  *% of mitotic type-1 cells* | 0.003 | 3 | 43.586 | <0.0001 | p16KO-CTL vs p16KO-RUN <0.0001  p16KO-CTL vs p16WT-CTL 0.0758  p16KO-CTL vs p16WT-RUN 0.0015  p16KO-RUN vs p16WT-CTL 0.0001  p16KO-RUN vs p16WT-RUN 0.0002 p16WT-CTL vs p16WT-RUN 0.2950 |
| Figure 3H  *Caspase-3 apoptotic cells* | <0.0001 | 3 | 9.495 | 0.0234 | p16KO-CTL vs p16KO-RUN 0.7603  p16KO-CTL vs p16WT-CTL 0.0212  p16KO-CTL vs p16WT-RUN 0.0212  p16KO-RUN vs p16WT-CTL 0.0001  p16KO-RUN vs p16WT-RUN 0.0464 p16WT-CTL vs p16WT-RUN 0.9999 |
